# Air pollution inequalities in Europe: A deeper understating of challenges in Eastern Europe and pathways forward towards closing the gap between East and West

**DOI:** 10.1097/EE9.0000000000000383

**Published:** 2025-04-24

**Authors:** Zorana Jovanovic Andersen, Artur Badyda, Lilian Tzivian, Angel M. Dzhambov, Katarina Paunovic, Stevan Savic, Bénédicte Jacquemin, Natasa Dragic

**Affiliations:** a Department of Public Health, University of Copenhagen, Copenhagen, Denmark; b Department of Informatics and Environment Quality Research, Faculty of Building Services, Hydro- and Environmental Engineering, Warsaw University of Technology, Warsaw, Poland; c Institute for Occupational, Social and Environmental Medicine, Centre for Health and Society, Medical Faculty and University Hospital Düsseldorf, Heinrich Heine University Düsseldorf, Düsseldorf, Germany; d Faculty of Medicine and Life Sciences, University of Latvia, Riga, Latvia; e Environmental Health Division, Research Institute at Medical University of Plovdiv, Medical University of Plovdiv, Plovdiv, Bulgaria; f Health and Quality of Life in a Green and Sustainable Environment Research Group, Strategic Research and Innovation Program for the Development of MU-Plovdiv, Medical University of Plovdiv, Plovdiv, Bulgaria; g Institute of Hygiene with Medical Ecology, Faculty of Medicine, University of Belgrade, Belgrade, Serbia; h Faculty of Sciences, Novi Sad Urban Climate Lab, University of Novi Sad, Novi Sad, Serbia; i Faculty of Natural Sciences and Mathematics, University of Banja Luka, Banja Luka, Bosnia and Herzegovina; j Univ Rennes, Inserm, EHESP, Irset (Institut de recherche en santé, environnement et travail), Rennes, France; k Faculty of Medicine, University of Novi Sad, Novi Sad, Serbia; l Institute of Public Health of Vojvodina, Novi Sad, Serbia

**What this study adds:**

In this commentary, we address the environmental inequalities in Europe, specifically related to air pollution exposure and related health burden disparities between Western and Eastern Europe. We first, provide a background for the causes of these inequalities and links with socioeconomic and demographic circumstances. We showcase, in more detail, air quality levels, monitoring, health research, and burden situation in four Eastern European countries (Serbia, Poland, Latvia, and Bulgaria), and briefly explain links with road traffic noise and climate change challenges in the region. At the end, we summarize several pathways and recommendations for going forward and overcoming air pollution exposure and the health burden gap in Europe.

## Background

**Figure 1.**
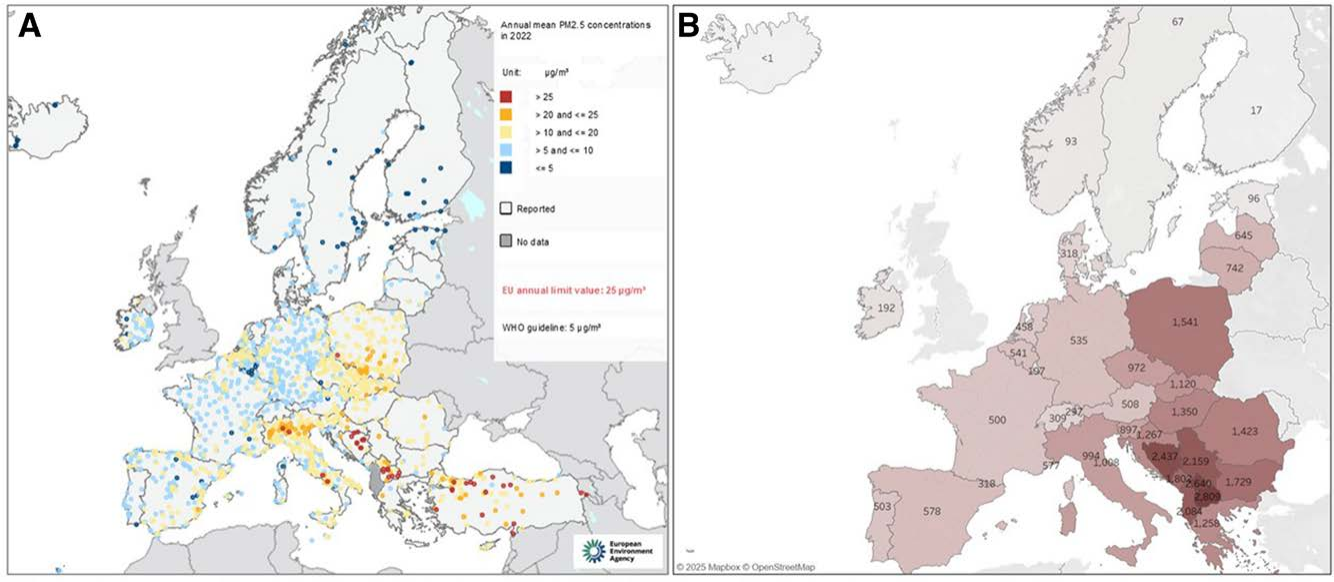
Concentrations of PM_2.5_ in 2022 from official EU (EEA) monitoring stations (37 countries) in relation to the EU limit value (25 µg/m^3^) (A). Number of deaths attributed to PM_2.5_ above WHO guidelines of 5 µg/m^3^ per country in 2022^1^ (B).

Air pollution is the biggest environmental stressor responsible for over 420,000 premature deaths in Europe in 2022,^1^ from cardiovascular diseases, chronic and infectious respiratory diseases, lung cancer, and type-2 diabetes.^2^ Air pollution levels in Europe have declined in the last decades, which has, at least in part, been attributed to successful European Union (EU) legislation. Ambient Air Quality Directive (AAQD)^3^ in 2008 set air quality standards (annual mean 25 µg/m^3^ for particulate matter with diameter <2.5 µm [PM_2.5_] and 40 µg/m^3^ for nitrogen dioxide [NO_2_]). These declines are most prominent in Western and Northern Europe, and there are still notable disparities in air pollution levels within and across countries in Europe,^4^ interlinked closely with socioeconomic inequalities (Figure 1 and Table 1). The Eastern European region, alongside Northern Italy, stands out as the most polluted in Europe, where many countries struggle to comply with current limit values and exceed many times World Health Organization (WHO) Air Quality Guidelines. The main air pollution sources in Eastern Europe are coal-dependent energy and outdated industry sectors, the widespread use of wood and coal for residential heating and cooking, old vehicle fleets, agricultural practices with high emissions of ammonia, poor waste management with industrial burning of waste materials, and open landfill waste burning still a common practice.^6^ These are rooted in poverty and socioeconomic and geopolitical systems dating back to the Soviet era, the civil war in the 1990s in former Yugoslavia and surrounding areas in the Balkans that has further destabilized the region, and similar current political instability and wars, such as Ukraine, all posing setbacks to economic development and access to clean energy. In Western Europe, major sources include traffic in urban areas, in lesser part local industry, energy production and wood burning for residential heating, and transboundary pollution. In Denmark, for example, more than 50% of PM_2.5_ comes from secondary sources from Eastern Europe. Another explanation for high pollution in Eastern Europe is that urbanization unfolded differently between the Eastern and Western regions, shaped by their unique historical, economic, and political paths. These differences have significantly influenced urban development patterns, socioeconomic conditions, and the environmental challenges each region faces. Key differences between Eastern and Western European stem from several factors: (1) economic disparities: Western Europe possesses greater financial resources to invest in climate adaptation, green infrastructure, and environmental initiatives, while Eastern Europe often depends on EU funding to modernize outdated systems; (2) regulatory frameworks: Western European countries have historically implemented stricter environmental policies and enforcement, whereas Eastern Europe has faced delays in aligning with EU standards due to transitional economies and governance challenges; (3) technological advancement: Western European cities lead in adopting smart technologies for pollution control, waste management, and urban planning, while Eastern Europe is still catching up; (4) cultural and historical factors: decades of industrial reliance and limited environmental awareness during the socialist era have left a legacy of pollution and inadequate infrastructure in Eastern Europe. Air pollution levels are up to several-fold higher in Eastern compared with Western countries (Figure 1), with the highest median PM_2.5_ in 2022 of 32, 30, and 23 µg/m^3^ measured in Bosnia and Herzegovina, Northern Macedonia, and Serbia, respectively, and lowest of 3, 5, 5, and 5 µg/m^3^ in Iceland, Estonia, Finland, and Sweden, respectively.^7^ As the EU has just in 2024 adopted new, much stricter air quality standards, in new revised AAQD with lower limit values of 10 µg/m^3^ for PM_2.5_ and 20 µg/m^3^ for NO_2_, and is preparing to revise other environmental and climate directives, there will be growing pressure on Eastern European governments to adopt new measures and speed up actions and policies to reach new standards. Corresponding to air pollution levels, Eastern European region suffers the highest air pollution-related health burden, with the highest rates of air pollution-related premature deaths^1^ (Figure 1) and cardiovascular diseases.^5^ This presents a major challenge for the EU to achieve a reduction in PM_2.5_-related premature deaths by 55% and the share of people disturbed by noise by 30% by 2030,^4^ as set out by EU Green Deal Zero Pollution Ambition.

**Table 1.** Illustrating differences between selected (coauthors’ countries of residence) Western and Eastern European countries in health, economic power, air pollution levels, health impacts, and sources

Country	Population in 2024	Life expectancy in 2022^a^	GDP^b^ in EUR per capita, 2022	PM_2.5_ (µg/m^3^), 2022^c^	PM_2.5-_related deaths/100,000 in 2022^d^
West					
Denmark	5,961,200	81.3	48,500	8	318
France	68,402,000	82.3	35,200	10	500
East					
Bulgaria	6,445,500	74.2	22,500	15	1,729
Latvia	1,871,900	74.5	25,000	10	645
Serbia	6,605,200	75.2	9,059	23	2,159
Poland	36,620,970	77.2	28,200	16	1,541
EU (27 members)	447,559,200	80.6	36,000	-	-

Sources: EUROSTAT:

a https://ec.europa.eu/eurostat/databrowser/view/demo_mlexpec/default/table?lang=en;

b https://ec.europa.eu/eurostat/databrowser/view/sdg_10_10__custom_12528861/bookmark/table?lang=en&bookmarkId=3b840ba1-9c24-41f2-88d6-ccea828df7ff;

c EEA^5^;

d EEA.^1^

In this commentary, we summarize the discussions from the workshop titled *“Environmental Justice in Europe: Closing the Gap in Air Pollution Health Effects Between East and West”* held at the International Society for Environmental Epidemiology Young Conference in Rennes, 5–7 June 2024. We highlight specific air pollution issues in four Eastern European countries—Bulgaria, Latvia, Serbia, and Poland—and compare them with two Western European countries, Denmark and France. These Western countries were selected due to their relevance to the coauthors, who are residents of these nations, and because they were mentioned during the workshop in discussions about the differences in air pollution between East and West (with Serbia being a non-EU member, and Poland, Latvia, and Bulgaria being EU member states). Additionally, we briefly explore the links between air pollution and other related environmental exposures, such as road traffic noise, which shares common sources with traffic-related air pollution. We also address the intersection of air pollution with climate change, as both are driven by greenhouse gas emissions—primarily from fossil fuel burning for energy production and residential heating. Finally, we outline potential strategies for reducing the air pollution gap between Eastern and Western Europe.

The countries selected for the workshop were chosen to reflect the geographical, cultural, environmental, political, and climatic diversity of Eastern Europe. This range spans from the colder, less polluted northern regions (Latvia) to the warmer southern countries (Serbia, Bulgaria). The group includes both older EU member states (Poland, Latvia) and newer members (Bulgaria), as well as a non-EU country (Serbia). The countries also represent a spectrum of pollution levels and associated health burdens, from the most polluted in Europe (Serbia) to those that have made progress in environmental improvements due to EU membership (Latvia, Bulgaria, and Poland).

## Showcasing air quality issues in four Eastern European countries

### Air pollution in Serbia

The latest report from the Serbian Environmental Protection Agency from 2023 concludes that air quality was category III (worst) for the majority of the largest cities in Serbia,^8^ with PM_10_ and PM_2.5_ as dominant pollutants. The annual mean of PM_10_ ranged from 14 to 60 µg/m^3^, with annual EU Directive 2008 and Serbian limit value (40 µg/m^3^) exceeded in nine of 25 cities, and daily limit value (50 µg/m^3^) exceeded in more than 60% of monitoring stations. The annual mean of PM_2.5_ ranged between 13 and 37 µg/m^3^ and exceeded the annual limit value (25 µg/m^3^) in 11 cities. The European Environmental Agency (EEA) report for the same year, based on the 13 monitoring stations from Serbia, showed PM_10_ concentrations ranging from 40 to 103 µg/m^3^, and for PM_2.5_ concentrations at five stations ranged between 10 and 20 µg/m^3^, between 20 and 25 µg/m^3^ in two stations, and was >25 µg/m^3^ at four stations.^7^ WHO air quality guidelines for PM_10_ and PM_2.5_, were exceeded at all monitoring stations in Serbia. Serbian national air quality monitoring network is quite comprehensive, with 96 stations in 2023,^8^ with 82 measuring PM_10_, 55 PM_2.5_, and 36 O_3_. National network is supplemented with local regional and municipality monitoring networks that cover all or some selected regulated pollutants. The question of availability and access to air quality data is, however, another issue. Data for national (automatic) network can be found online,^9^ but with irregular, sparse spatial and temporal coverage. WHO has in a special report on Serbia from 2019 recognized poor air quality, as well as important data limitations and gaps, calling for action.^10^

Epidemiological research on the health effects of air pollution is difficult due lack of air pollution models with appropriate temporal and spatial resolution, as well as the lack of cohorts or access to administrative health data for research. However, some local time-series studies have illustrated short-term air pollution impact on cardiovascular,^11^ cerebrovascular,^12^ and chronic obstructive pulmonary disease hospital admissions,^13^ as well as with all-cause mortality during the COVID-19 pandemic.^14^ Although there is a substantial collection of health and lifestyle data in Serbia, for example, the Serbian National Survey,^15^ Health Statistical Yearbook,^16^ and the National registers for diabetes, cancer, and acute coronary syndrome, these data are reported at an aggregate level, and not accessible at an individual level, nor linkable to each other, address data, etc., as needed for epidemiological research.

### Air pollution in Poland

Although air quality in Poland has been improving, it still ranks very poorly among EU countries. According to the EEA report^17^ and data from the Polish State Environmental Monitoring,^18^ in 2022, most pollutants in Poland met the EU air quality standards.^3^ Exceptions include daily PM_10_ levels and extraordinarily high concentrations of benzo(a)pyrene (BaP), for which the target concentration was met in only 21 of 165 monitoring stations. For daily PM_10_ concentrations in 2022, the median was 42 µg/m^3^, with the highest concentrations exceeding 80 µg/m^3^ (the limit value is 50 µg/m^3^), while annual limits (40 µg/m^3^) were met at all monitoring stations (median and maximum concentrations were 24 and 37 µg/m^3^, respectively). Minor and infrequent exceedances were also recorded for NO_2_ (with a median concentration at the level of 13 µg/m^3^) in several large cities. Although PM_2.5_ air quality standards are met in most locations (median concentration was 17 µg/m^3^ and the maximum 26 µg/m^3^), Poland remains the country with among the highest concentrations of this pollutant. WHO guidelines for PM_10_, PM_2.5_, and NO_2_, are exceeded at all stations.

Air quality monitoring system in Poland is quite well developed, with 284 monitoring stations, of which 213 are automatic or automatic-manual stations. Automatic measurement of PM_10_ is carried out in 178 stations and PM_2.5_ in 97 stations. The limitation of this system is the small number of traffic stations—only 20 in the whole country. In the face of the growing problem of traffic-related air pollutant emissions, especially in cities, this element of the monitoring system requires urgent expansion.

The municipal and residential heating sector plays a significant role in shaping air quality, responsible for 78.8% of PM_2.5_, 68.5% of PM_10_, and 53.5% of BC emissions in 2021.^17^ This sector is also the dominant source of polycyclic aromatic hydrocarbon emissions, with over 85% coming from solid fuel combustion. In cities, road transport significantly contributes to pollutant emissions, being a key source of NO_2_ and some metals. Nationally, NO_2_ emissions from transport account for nearly 32% of total emissions, but in large cities, this share can reach 45%.

The current air quality situation in Poland poses a serious threat to human health and life. The EEA report^7^ indicates that the number of years of life lost due to PM_2.5_ exposure alone in Poland is almost 520,000 and the number of deaths attributable to PM_2.5_, NO_2_, and O_3_ exceeds 53,000 annually. There are several local studies on the effects of short-term exposure to air pollution, which have links with asthma and chronic obstructive pulmonary disease hospital admissions,^19,20^ cardiovascular admissions,^21^ and total hospital admissions.^22^

Despite improvements in recent years, further radical reductions in the emission of air pollutants in Poland, especially from the residential sector, are necessary to enhance residents’ quality of life in the near future. In addition to the key contribution of this sector to PM emissions, a major air quality problem in Poland resulting from the widespread combustion of solid fuels (coal and wood) is the above-average high emission of benzo(a)pyrene, resulting in the highest concentration of this pollutant among all EU countries. In 2022, the median annual concentrations of this carcinogenic pollutant exceeded 2 ng/m^3^, with a maximum of almost 9 ng/m^3^, far higher than the WHO reference concentration (0.12 ng/m^3^) and the current EU target level (1 ng/m^3^).

Compliance with air quality standards will change dramatically after the new AAQD comes into force. The new standards for PM_10_ and PM_2.5_ would no longer be met at approximately 80% and 97% of monitoring stations, respectively. The situation for NO_2_ would also change, with exceedances potentially affecting about 15% of locations, compared with the current 2%. BaP levels would not be met at about 85% of monitoring stations.

### Air pollution in Latvia

The norms of air pollution in Latvia are defined by the Ministry Cabinet of Latvia. Air pollution in Latvia is monitored by the Latvian Centre for Environment, Geology, and Meteorology (LCEGM). Data provided by this authority are in line with the OECD report from 2019, and report on a pretty good air quality with a level of PM_2.5_ lower than OCED average (12.7 μg/m^3^ in Latvia versus 14 μg/m^3^ OECD average). However, the LCEGM reports are based on the measures from eight measuring stations; of them, four are located in small cities in different Latvian regions, and another four in the capital city Riga, which is the city with the largest population in Latvia (614,489 inhabitants in 2023). Of them, two stations are located in the park areas, one is located out of the city center, in the region where the density of vehicles is pretty low, and only one is located in the major road of the city. The number of measurement stations and their location raise concern about the quality of the provided data.

The information on air pollution provided by the LCEGM is based on traffic sources. However, traffic has a minor effect on air pollution in Latvia.^23^ Two major sources of air pollution are households and industry (56% and 30%, respectively), but information on the air pollution generated by these sources was not provided by LCEGM in their last report in 2021. According to the last LCEGM Latvia’s Informative Inventory Report (2021), the mean yearly concentrations in the small cities were 10.9 μg/m^3^ for PM_2.5_ and 18.9–22.7 μg/m^3^ for PM_10_. For Riga, the concentrations of PM in 2021 at the park stations were 13.9 μg/m^3^ for PM_2.5_ and 22.8 μg/m^3^ for PM_10_. Both stations located out of the park areas measure yearly and 24-hour concentrations of PM_10_, but not PM_2.5_. According to the data from the station at the city center, PM_10_ is constantly growing, reaching in 2021 the yearly level of 34.96 μg/m^3^ with 51 days when the level of PM_10_ exceeded 50 μg/m^3^.^24^

There are no local studies on air pollution health effects in Latvia, as there are no cohort studies or air pollution models. Moreover, the state seems to be not interested in providing real data for air pollution and monitoring it. For example, until 2016, there was another air pollution station located on one of the major streets in the Riga city center. This station showed the highest measures of air pollution, but it was closed in 2016 as a result of a traffic accident and was never repaired. With a very low number of studies and a lack of additional data on major sources of air pollution, the proper information on air quality in Latvia remains equivocal.

### Air pollution in Bulgaria

According to a report by the Ministry of Environment and Water, in the year 2022, the population was not exposed to PM_2.5_ above the annual mean exposure limit.^25^ The average exposure indicator (3-year running mean) for PM_2.5_ for 2022 was 16.23 µg/m^3^. However, 41% of the population was exposed to PM_10_ above EU standards. Annual mean PM_10_ exceedances were registered at 12 of 30 monitoring stations, with 74 of those in Plovdiv. In the Plovdiv agglomeration, the population was also exposed to annual mean NO_2_ above EU standards. According to the population-weighted annual means for PM_10_ (15 µg/m^3^) and NO_2_ (15.6 µg/m^3^) reported by the EEA, the WHO guidelines values for these pollutants exceeded in 2022. Bulgaria is also failing to meet its obligations to the EU for NH_3_ emission reduction, where agriculture contributes 93% of emissions. In 2022, household heating by burning solid fuel was the major source of PM_2.5_ (69%) and PM_10_ (47%), while coal-burning power plants contributed considerably to SO_2_ emissions (66%). The major source of NO_2_ was road transport (38%) followed by coal power plants (25%).

Air quality monitoring in the country is in line with current EU standards, but with 48 fixed monitoring stations (34 automated, nine manual samplings, five differential optical absorption spectroscopy), the spatial coverage is limited. Only about 10 of those measure PM_2.5_ and their positioning does not necessarily capture all relevant high-emission sites such as neighborhoods with high concentrations of domestic stoves used for household heating and smaller towns and settlements where the majority of the population uses such solid fuel appliances. Still, some municipalities have a local network of lower-class monitoring stations, such as Sofia where 22 sensors were installed within the pilot AIRTHINGS project (https://platform.airthings-project.com/). Recently, serious concerns have been raised about the validity of the data from these lower-class sensors.

Bulgaria faces similar challenges in air pollution and health as other countries. According to the EEA, in 2022, 9000 deaths were attributable to PM_2.5_, 1480 to NO_2_, and 930 to O_3_.^26^ Between 15% and 20% of the annual deaths from cardiovascular and respiratory diseases in Bulgaria can be attributed to air pollution, with the majority due to PM_2.5_ from residential heating and cooking on solid fuel and emissions from coal-burning power plants.^27^ Overall, virtually all Bulgarians are exposed to PM_2.5_ levels exceeding the WHO annual guideline value, and PM_2.5_ exposure is the main contributor to at least 11,000 death cases every year from air pollution (Bulgarian population 6.7 million people).^27^ That ranks air pollution the seventh leading risk factor for premature mortality in the country, though some cities like Plovdiv and Sofia, characterized by the abundance of emission sources and unfavorable topography and atmospheric circulation, have ranked as one of the top cities in the world in terms of PM_2.5_-related death rate.^27^ Additionally, many Bulgarians are energy-poor and rely on firewood and coal for heating their homes and cooking.^28^ Economic costs of these health impacts of air pollution are also substantial. Sofia’s losses in 2019 were estimated at 7.7%–13.4% of the city’s gross domestic product (GDP) or 2575–3124 billion euro.^29^

Air pollution and related co-exposures are exceedingly debated by non-governmental organizations, researchers, and medics, which have pushed for greater integration between sectors and the involvement of the state and local authorities in addressing air pollution-related health impacts.^27^ Air pollution and health research in Bulgaria has slowly been gaining momentum but studies still suffer from suboptimal air quality assessment techniques and lack of high-quality land-use regression models, limited data on some pollutants from sparsely distributed monitoring stations, and reliance on small, nonpopulation-based samples. More generally, there is a need for experts experienced in environmental epidemiology and a better exchange of knowledge and expertise between disciplines concerned with various aspects of the subject. Nevertheless, some notable positive developments include expanding the scope of research beyond respiratory health outcomes, mechanistic studies, and investigation of both short- and long-term effects of air pollution. Some recent projects include a representative health survey in Sofia that found higher odds of poor self-rated health with higher NO_2_ exposure,^30^ time-series analysis of cardiometabolic hospitalization of air pollution,^31^ and ongoing work on unraveling the role of the physical urban exposome in shaping health in a nationally-representative cross-sectional survey of over 4600 adults.^32^

## Air pollution, noise, and climate change in Eastern Europe

### Climate change

It is certain that the intensity of urbanization and traffic will increase in the next decades and amplify climate modifications in urban areas (particularly the air temperatures) compared with natural surroundings. Along with climate change crises and accelerating global warming, this will lead to more intensive urban heat island phenomena and higher heat-related health risks for populations. A recent report has found that the impact of rising temperatures in Europe is unequally distributed with more intense and longer heatwaves in Eastern and Southern Eastern Europe.^33^ Similarly, climate change predictions point out that Mediterranean regions are more susceptible to climate change, including some Eastern European countries.^34^ Additionally, exacerbated by already high air pollution levels, the health risks will accumulate after recent findings on the interaction of air pollution and heat, showing more air pollution-related cardiorespiratory deaths on days with high temperatures.^30,35^ In addition to rising temperatures and more frequent heatwaves, Eastern European cities are expected to experience a range of climate change-related extreme weather events that are closely linked to air pollution. These include increased ozone exposure due to higher temperatures and more sunlight, a rise in wildfire events and the associated pollution, and a greater frequency of storms and sandstorms that transport coarse particles from North Africa to European cities. Furthermore, more frequent droughts will lead to the resuspension of coarse dust particles in the air. The warmer climate will also introduce new aeroallergens and lengthen allergen seasons, exacerbating air quality and health impacts. To obtain more effective climate and health adaptation measures for cities, more complex assessments should be done that incorporate multiple segments of urban environments and include the interaction of urbanization-air pollution-urban climate.^36^ The impact of cities and urbanization on air quality is well known, but still interaction of urban climate and air quality should be investigated more deeply and monitored on local- and micro-scales.^37^

### Road traffic noise

Road traffic is one of the dominant sources of air pollution in urban areas, and also a source of another major stressor in urban areas, noise. Road traffic noise is the second biggest environmental stressor after air pollution, which is gaining growing interest from the research community and citizens.^38^ Over 20% of the EU population is exposed to harmful levels,^39^ resulting in annoyance, sleep disturbance, and 12,000 premature deaths.^38^ Noise levels remain stable or are increasing across Europe driven by economic growth-driven increases in car ownership. The noise problems are exacerbated in Eastern European cities many of which are struggling with high traffic congestion, have older and noisier vehicle fleets and public transport systems, low penetration of electric vehicles, and lack of investments in noise reduction measures. At the same time, there are poor noise data, apparent in the lack of noise monitoring in seven of 15 Eastern European countries. Lack of good quality noise mapping precludes awareness raising, research on the health effects of noise, and its interaction with air pollution, in Eastern European context. Therefore, in planning air pollution reduction measures in Eastern Europe, it is important to consider noise and prioritize solutions that effectively address both exposures.

## Pathways toward reduced air pollution inequality between Eastern and Western Europe

There are several barriers and priorities identified for moving forward to speed up air pollution reduction in Eastern and Southeastern Europe and reducing the gap between East and West.

First, need for better information about air pollution levels and composition and health-related risks in those specific regions. The Eastern European region is characterized by lower prioritization of environmental issues by governments, poor transparency, and lack of information about pollution, health risks, and risk mitigation measures, compared with Western Europe.^6^ Information campaigns targeted at all levels (policymakers, scientists, citizens, patients, medical professionals, and vulnerable groups) and citizen science projects can be invaluable in raising awareness and education of public on environmental issues, as a step towards building pressure on governments for implementing pollution reduction policies. In providing information, it is important to provide comprehensive information about air pollution as well as about related issues such as noise and climate change issues.

Second, need for local research and capacity building in environmental epidemiology. There is a general lack of local evidence on the health effects of air pollution and related exposures, due to poor research infrastructure (lack of data on exposure or health, or both), and lack of investments in research and training in public and environmental health.^6^ As knowledge from Western Europe is not directly transferrable to Eastern Europe due to differences in air pollution sources and socioeconomic, demographic, and health backgrounds of the populations, there is a need for local capacity building in data and research staff. Local studies are urgently needed to fully grasp the impact of air pollution, noise, and climate impacts on health in Eastern Europe. They are also invaluable for building local capacities, raising awareness of local communities, and supporting evidence-based decision-making. Local capacity building can be efficiently accomplished by research collaboration between Western and Eastern researchers and institutions, sharing of knowledge, and know-how, to build capacity and develop sustainable infrastructure for research in Eastern Europe.

Third, engaging policymakers with citizen needs. In addition to the lack of evidence for shaping policies in Eastern Europe, it often happens (in East and West) that policy recommendations are disconnected from people’s daily lives and needs. Policymakers have little insight into the social, cultural, behavioral, and economic contexts and experiences of people most affected by air or noise pollution, and motivating drivers of change among these groups, which is why policies fail to generate positive change. At the same time, as the economy and standard of living are improving in Eastern European countries, citizens are increasingly receptive to environmental issues and demand clean air actions and general cleaner environmental standards from their governments. This is a unique opportunity to bridge the gap between policy and people and use cocreation to solve air pollution problems, from local neighborhoods to city, national, and regional levels. Cities can play an important role in engagging with citizens to cocreate solutions integrating air pollution reduction measures in urban planning, greening measures, climate action plans, traffic planning, etc.

Fourth, foster better collaboration between Western and Eastern European countries and cities in sharing experiences on successful air pollution reduction policies and actions, which can be easily transferred from West to East. Poor air quality in Eastern Europe is not just a “local” problem. Pollution is transported over long distances affecting neighboring EU countries and entire regions. For example, in Denmark, up to 50% of PM_2.5_ comes from long-range transported particles from Central and Eastern Europe,^40^ illustrating the need for a coordinated local, national, and international collaboration to achieve zero air pollution across Europe.

Fifth, at the EU level, it is crucial to prioritize policies and measures that accelerate, rather than delay, air pollution reductions in Eastern Europe to reduce inequalities and close the gap between East and West. The EU must provide the necessary financial support to Eastern European countries with the lowest GDP to help them transition to cleaner energy production, phase out coal, adopt cleaner industrial practices, eliminate the use of solid fuels for residential heating and cooking, renovate and insulate homes, and develop public transportation and active travel infrastructure in cities. Additionally, it is important to encourage local policymakers to support these initiatives by demonstrating that the long-term economic benefits, including savings in healthcare costs, will far outweigh the initial investment and contribute to sustained growth and prosperity.

## Conclusions

While air quality has improved in Europe over the past few decades, significant disparities remain between East and West that demand urgent action. Eastern European citizens continue to breathe the most polluted air, and suffer the highest air pollution health burden—up to several times higher air pollution mortality rates than in Western European countries. This large air pollution burden inequality in Europe is unacceptable and reducing and eliminating it should be one of the key priorities in implementing EU Green Deal Zero Pollution Goals. At the 2024 International Society for Environmental Epidemiology workshop in Rennes, we discussed the air pollution situation with experts from four Eastern European countries (Latvia, Serbia, Poland, and Bulgaria) and workshop participants and identified key actions to address the air pollution gap in Europe, and provide clean air for all: (1) improve data and access to information on air pollution exposure and health effects for citizens in Eastern Europe; (2) invest in research and capacity building in environmental epidemiology to generate local evidence on the health impacts of air pollution, as well as related exposures like noise, climate change factors (e.g., heatwaves, wildfires), and their interactive effects; (3) involve policymakers and citizens in cocreating policies and measures to reduce pollution; (4) foster collaboration between Western and Eastern European countries and cities to share successful governance models for air pollution reduction; (5) at the EU level, prioritize policies that accelerate air pollution reductions in Eastern Europe and provide the necessary financial support for their effective implementation.

## Conflicts of interest statement

The authors declare that they have no conflicts of interest with regard to the content of this report.
